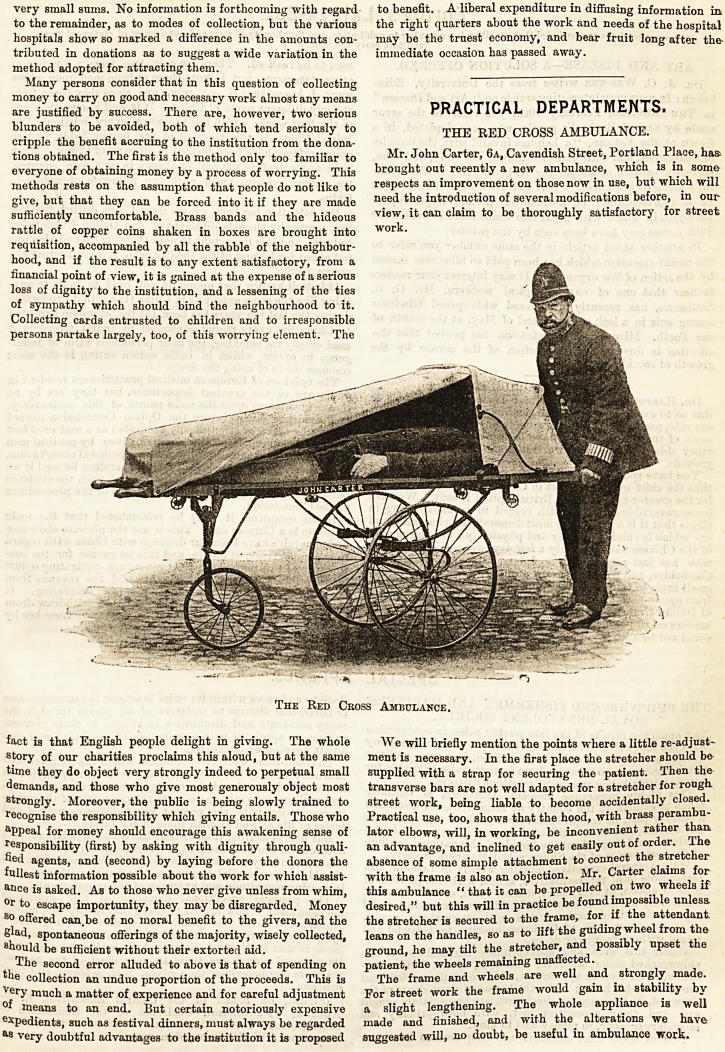# The Red Cross Ambulance

**Published:** 1894-02-24

**Authors:** 


### PRACTICAL DEPARTMENTS.

### THE RED CROSS AMBULANCE.

Mr. John Carter, 6A, Cavendish Street, Portland Place, has brought out recently a new ambulance, which is in some respects an improvement on those now in use, but which will need the introduction of several modifications before, in our view, it can claim to be thoroughly satisfactory for street work.

THE RED CROSS AMBULANCE.

We will briefly mention the points where a little re-adjustment is necessary. In the first place the stretcher should be supplied with a strap for securing the patient. Then the transverse bars are not well adapted for a stretcher for rough street work, being liable to become accidentally closed. Practical use, too, shows that the hood, with brass perambulator elbows, will, in working, be inconvenient rather than an advantage, and inclined to get easily out of order. The absence of some simple attachment to connect the stretcher with the frame is also an objection. Mr. Carter claims for this ambulance "that it can be propelled on two wheels if desired," but this will in practice be found impossible unless the stretcher is secured to the frame, for if the attendant leans on the handles, so as to lift the guiding wheel from the ground, he may tilt the stretcher, and possibly upset the patient, the wheels remaining unaffected.

The frame and wheels are well and strongly made. For street work the frame would gain in stability by a slight lengthening. The whole appliance is well made and finished, and with the alterations we have suggested will, no doubt, be useful in ambulance work.

## Figures and Tables

**Figure f1:**